# Epstein-Barr virus *mir-bart1-5p* detection via nasopharyngeal brush sampling is effective for diagnosing nasopharyngeal carcinoma

**DOI:** 10.18632/oncotarget.6649

**Published:** 2015-12-18

**Authors:** Xiao-Hui Zheng, Li-Xia Lu, Cui Cui, Ming-Yuan Chen, Xi-Zhao Li, Wei-Hua Jia

**Affiliations:** ^1^ Sun Yat-Sen University Cancer Center, State Key Laboratory of Oncology in South China, Collaborative Innovation Center for Cancer Medicine, Guangzhou 510060, China; ^2^ Department of Experimental Research, Sun Yat-Sen University Cancer Center, Guangzhou 510060, China

**Keywords:** nasopharyngeal carcinoma, Epstein-Barr virus, microRNA, nasopharyngeal brush, biomarker

## Abstract

Epstein-Barr virus (EBV)-encoded microRNAs (miRNAs) are highly expressed in nasopharyngeal carcinoma (NPC) cases in high-risk areas, and may be involved in tumorigenesis. Using quantitative RT-PCR, we detected four EBV-encoded BamHI A rightward transcript (BART) miRNAs (*mir-bart1-5p*, *mir-bart5*, *mir-bart6-5p* and *mir-bart17-5p*) exclusively in 53 NPC biopsies as compared to 69 controls. In a larger patient group, that included 215 NPC cases and 209 controls, significantly higher levels of all four EBV miRNAs were detected in tumor cells harvested directly from the nasopharynx using a less invasive nasopharyngeal (NP) brush than in the controls (p < 0.001). One EBV miRNA, *mir-bart1-5p,* holds particular promise for use as a diagnostic indicator of NPC (with 93.5% sensitivity and 100% specificity), and its relative expression level was reflective of disease progression. Detection of this miRNA was effective for diagnosing early-stage NPC, even in cases that were falsely diagnosed as negative based on histopathological analysis, plasma EBV DNA load, and VCA-IgA and EA-IgA titers. EBV-encoded *mir-bart1-5p* detection via NP brush sampling could act as an efficient and less invasive method assisting clinical diagnosis of NPC.

## INTRODUCTION

Nasopharyngeal carcinoma (NPC) is a highly invasive and metastatic cancer that is widely prevalent in southern China and Southeast Asia. Although the overall survival rate is approximately 90% in patients with early stage disease, most patients are diagnosed with advanced disease at their first clinic visit, and the survival rate for these patients is less than 50% [1]. Developing a simple and reliable method for diagnosing and monitoring NPC is of great importance for improving patient outcomes [2].

The infection of epithelial cells by Epstein-Barr virus (EBV) is a typical characteristic of NPC cases in high-risk areas [3, 4]. Therefore, substantial effort has been made to develop novel EBV detection approaches utilizing EBV-related markers. Methods for detecting antibody titers against EBV serum antigens, including viral capsid antigen (VCA) and early antigen (EA), and for detecting EBV DNA load in plasma have been developed to assist in NPC diagnosis [5–8]. However, the results of antibody titer tests alone have proven insufficient to accurately diagnose NPC [5]. Additionally, EBV DNA tests appear to be of limited value for diagnosing NPC patients with early stage disease and local recurrence [8].

As an alternative to the more-invasive biopsy method, tumor tissue samples can also be obtained directly from the nasopharynx using a nasopharyngeal (NP) brush or swab. Samples from NP brushes or swabs are considered to directly reflect the local tumor load and disease process. EBV DNA is a popular biomarker [9–16] based on the hypothesis that normal NP tissue is negative for EBV [17]. However, evidence from endemic areas suggests that EBV DNA is also present in samples from normal NP tissue [12, 14, 15]. Our recent study showed that EBV DNA was detectable in 70.6% of NP brush samples from non-NPC controls and in 87.8% of samples from high-risk controls [16]. Another recent study detected significantly elevated EBV DNA loads in NP brush samples from patients with non-NPC head and neck tumors [15].

Previous studies have also demonstrated that a series of EBV-encoded microRNAs (miRNAs) are specifically expressed in NPC tumors, particularly the BamHI A rightward transcript (BART) miRNAs, and may play a role in tumorigenesis [18]. Because EBV gene products should only originate from infected cells, we hypothesized that detecting functional EBV miRNAs could improve the accuracy and efficiency of NPC diagnosis. miRNAs located in the EBV cluster 1 region reportedly exhibit higher expression and lower degrees of variability than miRNAs from the cluster 2 region [18–20]. Five miRNAs, including *mir-bart1-5p*, *mir-bart5*, *mir-bart6-5p*, *mir-bart16* and *mir-bart17-5p,* were selected for testing in our study. However, *mir-bart16* was excluded due to its relatively poor performance in our preliminary experiments (data not shown). The aim of this study was to assess the value of detecting EBV miRNAs from NP brush samples for diagnosis and monitoring of NPC.

## RESULTS

### Characteristics of the study population

**Figure 1:**
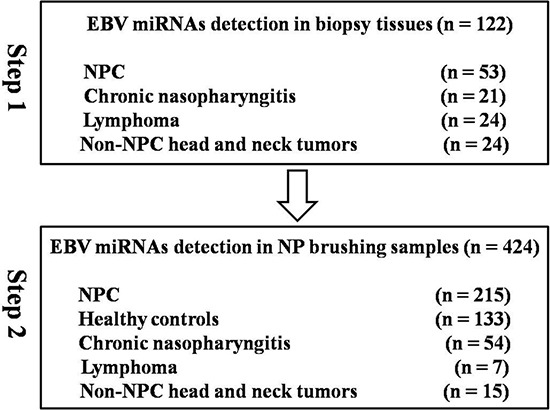
Study workflow

Biopsy tissue samples were collected from 122 different patients, including 53 NPC patients, 21 with chronic nasopharyngitis, 24 with lymphoma and 24 with non-NPC head and neck tumors (Figure 1). Samples from patients with lymphoma or non-NPC head and neck tumors were not taken from the nasopharynx region.

NP brush samples were collected from 424 different participants. There was no significant difference in age (p = 0.426) or gender (p = 0.095) between case and control groups. In contrast, significant differences in VCA-IgA titer (P < 0.001) and EA-IgA titer (P < 0.001) were observed. TNM staging was performed for the patients using the Chinese 2008 staging system. Additionally, nearly all of the patients were classified as WHO type 3. The detailed characteristics of the study population are presented in Table 1.

**Table 1:** Characteristics of participants undergoing nasopharyngeal brush sampling

Variable	NPC	Controls	P value
**Number**	215	209	
**Age(y)**			0.426
mean	47.1	47.8	
**Gender**			0.095
male	157	137	
female	58	72	
**VCA-IgA titer**			<0.001
negativity	20	129	
1:10 - 1:40	21	50	
≥1:80	174	30	
**EA-IgA titer**			<0.001
negativity	67	195	
1:10 - 1:40	134	14	
≥1:80	14	0	
**Cancer stage^#^**			
Stage I/II	15		
Stage III	86		
Stage IV	48		
**T stage**			
T1/T2/T3/T4	7/28/81/33		
**N stage**			
N0/N1/N2/N3	6/59/70/14		
**M stage**			
M0/M1	137/12		
**Histopathology**			
WHO 3	2/213		

# The pathological staging information was evaluated by clinical doctors based on comprehensive results of magnetic resonance (MR), histopathology and clinical symptoms. Some patients were biopsy-diagnosed with NPC in our Cancer Center but subsequently moved to other hospitals for further diagnosis and treatment. For these patients, results such as MR were not collected, while only their histopathology information was obtained. The staging information of 66 patients was lost in this study.

### EBV miRNAs were uniquely expressed in biopsy tissues from NPC patients

**Figure 2:**
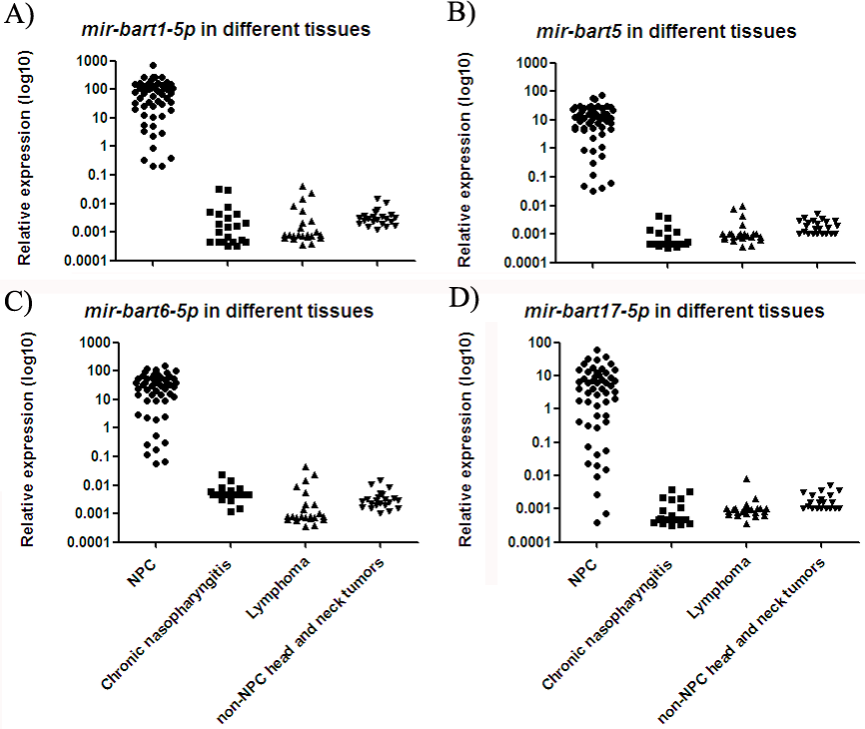
Expressions of four EBV miRNAs in different NP tissue samples The levels of *mir-bart1-5p* **A.** *mir-bart5* **B.** *mir-bart6-5p* **C.** and *mir-bart17-5p* **D.** were higher in tissue samples from NPC patients as compared to non-NPC controls (p < 0.001).

Four EBV miRNAs (*mir-bart1-5p*, *mir-bart5*, *mir-bart6-5p* and *mir-bart17-5p*) exhibited high expression in biopsy tissue samples from NPC patients, whereas they were only detected at low levels in non-NPC controls (Figure 2). The median expression values of *mir-bart1-5p, mir-bart5, mir-bart6-5p* and *mir-bart17-5p* were 68, 11, 33 and 4.1, while their median expression values in non-NPC controls were 0.002, 0.001, 0.003, and 0.001, respectively. The fold changes of the four miRNAs were 22220, 10255, 7869 and 5416 (all with p < 0.001). The relative expression ranges of the miRNAs in clinical tissue samples are presented in Supplementary Table S1.

### EBV miRNAs were significantly elevated in nasopharyngeal brush samples from NPC patients

**Figure 3:**
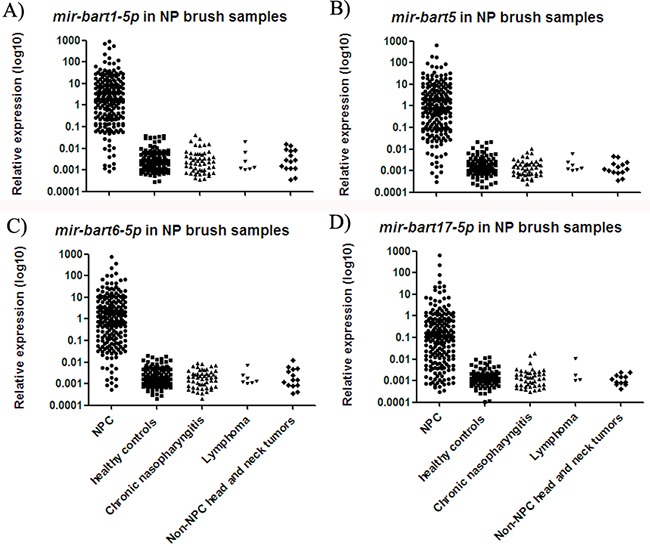
Expressions of four EBV miRNAs in NP brush samples from different participants The levels of *mir-bart1-5p* **A.** *mir-bart5* **B.** *mir-bart6-5p* **C.** and *mir-bart17-5p* **D.** were significantly increased in the NPC group relative to the controls (p < 0.001).

NP brush sampling provided a less invasive pathway for directly collecting samples from the nasopharynx (Supplementary Figure S1). Similar to the results for biopsy tissue samples, relative expression of all four miRNAs was significantly increased in NP brush samples from NPC patients as compared to controls (p < 0.001; Figure 3). The median expression values of *mir-bart1-5p, mir-bart5, mir-bart6-5p* and *mir-bart17-5p* were 2.05, 0.84, 1.08 and 0.12, while their median expression values in controls were 0.002, 0.001, 0.002, and 0.001, respectively. The fold changes were 4300, 4840, 4350 and 3700 (all with p < 0.001). The relative expression ranges of the miRNAs in the NP brush samples are presented in Supplementary Table S2.

### EBV miRNAs from NP brush samples can be used as biomarkers for diagnosing NPC

Receiver operating characteristic (ROC) analysis revealed that *mir-bart1-5p, mir-bart5, mir-bart6-5p* and *mir-bart17-5p* were all valuable biomarkers for differentiating NPC from controls. Area under the curve (AUC) values were 0.98 (95% CI, 0.96 to 0.99), 0.97 (95% CI, 0.95 to 0.99), 0.97 (95% CI, 0.94 to 0.99) and 0.92 (95% CI, 0.89 to 0.95), respectively. At the cut off value (COV) of 0.050 for *mir-bart1-5p*, the optimal sensitivity and specificity were 93.5% and 100%, respectively. At the COV of 0.023, the optimal sensitivity and specificity for *mir-bart5* were 91.2% and 100%; for *mir-bart6-5p* these values were 91.6% and 100%, respectively. *mir-bart17-5p* exhibited slightly less accuracy, with 83.7% sensitivity and 92.8% specificity (Table 2). Combining the four miRNAs did not strengthen diagnosis accuracy (data not shown). The correlation coefficients of *mir-bart1-5p* with *mir-bart5*, *mir-bart6-5p* and *mir-bart17-5p* were 0.93, 0.97 and 0.84, respectively (all with p < 0.001; Supplementary Figure S2).

**Table 2:** Sensitivity, specificity, PPV and NPV of four EBV miRNAs

		Case		Control						
miRNAs	COV	Median expression	No. positivity/negativity	Median expression	No. positivity/negativity	Sensitivity (%)	Specificity (%)	PPV (%)	NPV (%)	AUC
mir-bart1-5p	0.050	2.05	201/14	0.002	0/209	93.5	100	100	93.7	0.98
mir-bart5	0.023	0.84	196/19	0.001	0/209	91.2	100	100	91.6	0.97
mir-bart6-5p	0.023	1.08	197/18	0.002	0/209	91.6	100	100	92.1	0.97
mir-bart17-5p	0.004	0.12	180/35	0.001	15/194	83.7	92.8	92.3	84.7	0.92

PPV: positive predictive value

NPV: negative predictive value

### *mir-bart1-5p* levels in NP brush samples were reflective of disease progression

**Figure 4:**
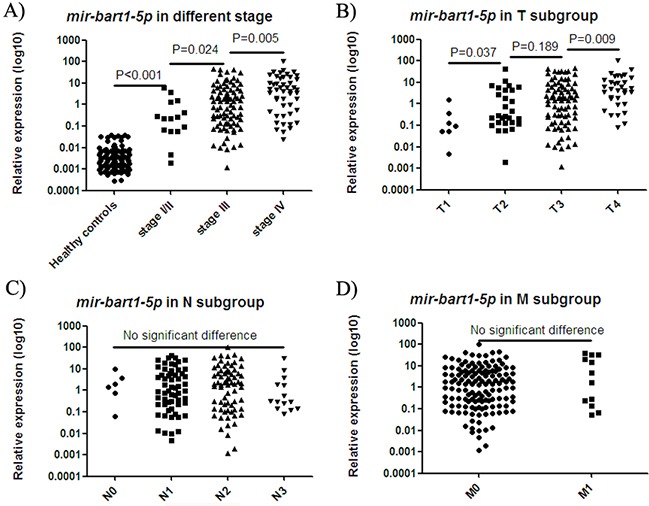
mir-bart1-5p expression in samples at various stages of disease progression The expression of *mir-bart1-5p* in the NP brush samples of healthy controls was significantly lower than in stage I/II NPC samples **A.** and samples from the T1 subgroup **B.** Stage I/II A. and T1 subgroup B. NPC samples also exhibited significantly lower levels of EBV *mir-bart1-5p* compared with the levels observed in samples from more advanced disease. No significant difference was observed in node–positive/negative **C.** or metastasis-positive/negative cases **D.**

Due to its high correlation with the other three miRNAs, *mir-bart1-5p* was further analyzed in samples at various stages of disease progression. Levels of *mir-bart1-5p* in control NP brush samples were significantly lower than in stage I/II NPC samples (p < 0.001; Figure 4A), and samples from the T1 subgroup (p < 0.001; Figure 4B). Stage I/II and T1 subgroup NPC samples also exhibited significantly lower levels of EBV miRNAs when compared with samples from patients with more advanced disease. In contrast, no significant difference was observed in node–positive/negative or metastasis-positive/negative cases (Figure 4C and 4D).

### Diagnosis by *mir-bart1-5p* versus pathological biopsy, VCA-IgA and EA-IgA

We further compared the diagnostic performance of *mir-bart1-5p* with common diagnostic methods, including histopathological assessment based on initial biopsy sampling, and molecular diagnosis based on VCA-IgA and EA-IgA detection.

In this study, repeated biopsy sampling was conducted in 16 patients. The expression of *mir-bart1-5p* was above the COV (0.050 copy/RUN6B) in only the NP brush samples. The results of diagnoses by biopsy and *mir-bart1-5p* in NP brush samples are summarized in Supplementary Table S3. Computed tomography (CT) and magnetic resonance imaging (MRI) results, along with endoscopy images, are shown for a representative patientin Supplementary Figure S3. A slightly higher sensitivity (93.5%) was obtained using *mir-bart1-5p* detection as compared with initial biopsy (92.6%; Table 3).

**Table 3:** Diagnostic performance of *mir-bart1-5p* and other current methods

	COV	Sensitivity (%)	Specificity (%)	PPV (%)	NPV (%)
mir-bart1-5p	0.050	93.5	100	100	93.7
Initial biopsy	N/A	92.6	100	100	92.9
VCA-IgA	≥ 1:80	80.9	85.6	85.3	81.3
EA-IgA	≥ 1:10	68.8	92.8	91.4	74.4

IgA antibody titers for VCA-IgA and EA-IgA are traditional markers used in areas at high-risk for NPC. VCA-IgA exhibited 80.9% sensitivity and 85.6% specificity at COV ≥ 1:80, whereas EA-IgA exhibited 68.8% sensitivity and 92.8% specificity at COV ≥ 1:10 in this study (Table 3).

## DISCUSSION

Early and accurate diagnosis of NPC is important for enhancing patient survival. Blood and NP brush tissue-sampling methods, which are less invasive than biopsies, show promise for diagnosing and monitoring NPC through detection of EBV-related biomarkers (such as VCA-IgA titer and EBV DNA load). In this study, detection of EBV *mir-bart1-5p* in NP brush samples was identified as a novel method for clinically diagnosing and monitoring the progression of NPC. The clinical application of a NP brush sampling/EBV miRNA detection method could help to improve patient outcomes in high-prevalence areas such as southern China.

Utilizing NP brush sampling to detect EBV DNA load has attracted increasing attention in recent years, and various DNA fragments have been detected in multiple studies. For example, a 213-bp region of EBV nuclear antigen-1 (EBNA-1) [14, 15] and a 75-bp fragment of the EBV BamHI-W region [12] are commonly used in detection studies, and have comparable sensitivities and specificities. However, EBV DNA has also been detected in NP brush samples from patients with non-NPC head and neck tumors [15]. EBV-encoded small RNA (EBER) *in situ* hybridization (ISH) is a useful qualitative technique for identifying EBV infection in cells [21]. EBER ISH results from this study showed that high EBER signaling was only observed in tissue slices from NPC patients, suggesting there was no EBV infection in non-NPC controls (Supplementary Figure S4). Similarly, we observed that the EBV miRNAs tested in this study (*mir-bart1-5p, mir-bart5 and mir-bart6-5p*) were detected only in NPC patient samples, and not in any of the controls (Figure 3), resulting in a specificity of 100% for NPC diagnosis (Table 2). These results verified our hypothesis that quantitative detection of functional EBV miRNA transcripts could be used to effectively diagnose NPCs. Specific EBV miRNA detection could also potentially be used in combination with EBER ISH to further enhance NPC diagnosis accuracy.

Plasma EBV DNA testing appears to be of limited value when diagnosing patients with early clinical stage NPC [22] or local recurrence [23]. Small tumors may release only low levels of EBV DNA fragments into the blood. By contrast, our findings indicate that EBV miRNAs can be effectively used to diagnose early stage NPC. EBV *mir-bart1-5p* detected in NP brush samples was used to correctly diagnose 13 NPC cases at stage I/II and 6 cases at T1 in the first sampling. The potential value of EBV *mir-bart1-5p* for early NPC diagnosis is further illustrated by the following two cases. First, a 49-year-old man presented with minor symptoms and was negative for plasma EBV DNA load and for VCA-IgA and EA-IgA titers. The second patient was a 74-year-old man with a VCA-IgA titer of 320. The histopathological assessments based on initial NP biopsy were NPC-negative for both patients, likely due to no obvious tumor mass under endoscopic examination. The two patients respectively underwent four and two biopsies before a correct pathological diagnosis was achieved. The first patient was diagnosed with T1N2M0, stage III disease, and the second patient was diagnosed with T1N2M1, stage IV disease. In contrast to the other diagnostic techniques used, expression of *mir-bart1-5p* at initial NP brush sampling was above COV (0.05 copy/RUN6B) in both cases, indicating its unique diagnostic value even for early NPC cases.

We also observed that *mir-bart1-5p* expression potentially could be used as a biomarker for monitoring NPC progression, in that increasing *mir-bart1-5p* expression correlated with disease progression (Figure 4A). However, expression was only significantly correlated with the T subgroup of NPC (Figure 4B), not N and M stage (Figure 4C and 4D). NP brush sampling may directly reflect the local tumor load in the nasopharynx.

The nasopharynx is often difficult to access, and pathological diagnosis based on biopsies may lead to false negatives. While initial biopsy sampling resulted in a diagnostic sensitivity of 92.6% among patients in this study (Table 3), 16 out of 215 NPC patients required repeated biopsy sampling in order to obtain a correct pathological confirmation. Notably, the EBV *mir-bart1-5p* levels in the NP brush samples from these 16 patients were all above the COV, correctly indicating the presence of NPC (Supplementary Table S3). Similarly, in nearly all cases for which VCA-IgA titer produced a false negative diagnosis (38 out of 41), *mir-bart1-5p* expression was above the COV (data not shown).

Still, there were 14 NPC cases in which NP brush samples tested negative for *mir-bart1-5p* expression. Most of these cases were diagnosed within the T3 subgroup. *mir-bart1-5p* detection and EBER ISH were both conducted directly with tissue slices from these patients. The results showed *mir-bart1-5p* and EBERs were both expressed in nasopharyngeal tumor cells (Supplementary Figure S5). It is possible that NP brush sampling failed to capture tumor cells in these samples, leading to false negative results. Although repeated brush sampling is feasible, further optimization of the sampling method is required. For example, a brush with a shape more suitable to the physiological structure of the nasopharynx should be developed.

In conclusion, quantification of EBV *mir-bart1-5p* in NP brush samples could be used as an effective supplementary method for NPC diagnosis. Thanks in part to the reduced invasiveness of the described NP brush sampling/EBV miRNA detection NPC diagnostic technique, it has great potential for use in monitoring disease progression in NPC patients and in the screening of high-risk populations.

## MATERIALS AND METHODS

### Clinical specimens and study design

To identify EBV miRNAs expressed exclusively in NP tissues from NPC patients, 122 clinical tissue samples from patients with chronic nasopharyngitis, lymphoma, non-NPC head and neck tumors, and NPC were obtained from the Tumor Resource Bank of our cancer center. Subsequently, a total of 424 participants undergoing NP brush sampling were recruited from January 2013 to October 2015. Two hundred and ninety one NP brush samples were consecutively collected from participants when they underwent NP biopsy at Sun Yat-Sen University Cancer Center (SYSUCC). Among the participants, 215 exhibited biopsy-positive NPC, 54 were eventually diagnosed with chronic nasopharyngitis, 7 were diagnosed with lymphoma, and 15 had non-NPC tumors. Additionally, 133 NP brush samples from healthy control individuals were collected from Sihui city, a high-risk area of Guangdong province, China. A three ml blood sample was collected from each participant. The Human Ethics Committee of the Sun Yat-Sen University Cancer Center approved this study, and all participants provided informed consent.

### Sampling procedures

Experienced specialists and resident trainees conducted NP brush sampling under the guidance of endoscopy. An endoscope was used to evaluate the entire nasopharynx, and images were captured at the site of suspicious tumors. Before biopsy, a NP brush (Copan Diagnostics) was inserted into the nose until the NP cavity was reached. The brush was rotated several times over the NP epithelium at the site of the suspected lesion and then quickly removed. Immediately after sampling, the brush tip (1.5 cm) was cut, placed in one ml of RNAlater (Invitrogen, Carlsbad, CA, USA) and stored at −80°C until use. For healthy participants, NP brush samples were obtained from the lateral pharynx because this was the most common site for disease occurrence.

### RNA extraction, reverse transcription (RT) and quantitative polymerase chain reaction (Q-PCR)

Total RNA from biopsy tissue and NP brush samples was extracted using the TRIzol reagent (Invitrogen, Carlsbad, CA, USA) according to the manufacturer's instructions. The RNA concentration was quantified using a NanoDrop 1000 Spectrophotometer (NanoDrop Technologies, Waltham, MA).

A total of 350 ng RNA (100 ng in tissue samples) was used for reverse transcription with a TaqMan MicroRNA Reverse Transcription Kit (Applied Biosystems, Foster City, CA, USA) according to the manufacturer's protocol. Reverse transcription reactions (20 μl volume/sample) were conducted with custom stem-loop primers (Applied Biosystems) specific to the corresponding mature sequence obtained from miRBase (www.miRBase.org). RNU6B was usually used as the endogenous control, and 4 EBV miRNAs were targets. Each reaction contained 0.2 μl dNTPs (100 mM), 1.3 μl MultiScribe Reverse Transcriptase (50 U/μL), 2.0 μl 10X Reverse Transcription Buffer, 0.25 μl RNase Inhibitor (20 U/μL), 8 μl RT primer (1.6 μl/per primer) and 8.25 μl RNA and nuclease-free water (350 ng total RNA). The following parameter values were used to perform the thermal cycles: 16°C for 30 min, then 42°C for another 30 min followed by 85°C for 5 min.

Q-PCR was carried out on the LightCycler®480 instrument (Roche). Each PCR reaction (10 μl volume) included 1 μl of RT product, 5 μl of TaqMan 2X Universal PCR Master Mix, 0.5 μl of 20X TaqMan MicroRNA Assay mix and 3.5 μl nuclease-free water. All reactions were performed in duplicate and the relative expression of each miRNA (copy number/RNU6B) in one sample was determined using the equation 2^−ΔCT^, where ΔCT = Ct (miRNA) – Ct (RNU6B) (Ct value were the threshold cycle for each miRNA). Thermal cycling was initiated with a denaturation step of 10 min at 95°C, followed by 40 cycles of 95°C for 15 s and 60°C for 1 min.

### VCA-IgA and EA-IgA antibody titers in blood specimens

The VCA-IgA and EA-IgA titer of EBV were measured using a commercial kit (Zhongshan Bio-tech Co Ltd., Zhongshan City, China) based on standard techniques described previously [24].

### EBERs and *mir-bart1-5p* in situ hybridization

Paraffin-embedded tissue sections were obtained from the Pathology Department of our cancer center. They included 14 NPC samples with negative *mir-bart1-5p* expression in NP brushing samples, 7 lymphoma samples, 3 non-NPC other tumor samples and 16 NP tissue samples with chronic nasopharyngitis. The presence of EBV in tumor cells was assessed by in situ hybridization (ISH) using the EBER ISH kit (ZSGB Bio-tech Co Ltd., Beijing, China). A DIG-double labeled locked nucleic acid (LNA)-based probe specific for *mir-bart1-5p* (Exiqon, Vedbaek, Denmark) was used to detect *mir-bart1-5p* in tumor cells. ISH was performed according to the manufacturer's instructions.

### Statistical analyses

The Mann-Whitney test was used to compare the nonparametric variables between NPC and control groups. A one-way ANOVA was used to compare the differences in multiple groups. Differences in a demographic variable (e.g., sex) between the cases and all controls were evaluated using a Chi-square test. EBV miRNA correlations were assessed by applying Pearson correlation coefficients and were subjected to two-tailed significance tests; p < 0.05 was considered significant. Receiver operating characteristic (ROC) curves were used to evaluate diagnostic value, and area under the ROC curve (AUC) was used as an accuracy index for evaluating diagnostic performance. We used the curves to select cut-off values (COV) with maximum sensitivity and specificity. The statistical analyses were performed using the SPSS 16 software.

## SUPPLEMENTARY FIGURES AND TABLES


