# Supplementary material for: Epstein-Barr virus mir-bart1-5p detection via nasopharyngeal brush sampling is effective for diagnosing nasopharyngeal carcinoma
Source: Oncotarget. 2015 Dec 18;7(4):4972–80. doi: 10.18632/oncotarget.6649 (PMC4826258; doi:10.18632/oncotarget.6649)
Supplement: Supplementary file 1 [file oncotarget-07-4972-s001.pdf]

SUPPLEMENTARY FIGURES AND TABLES

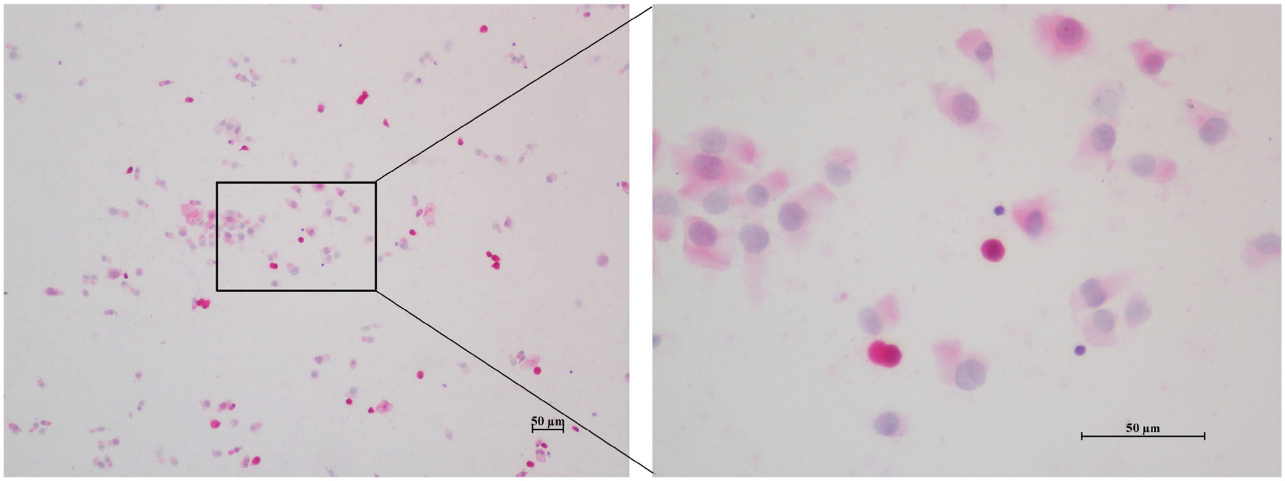

Supplementary Figure S1: A typical NP brush smear was conducted. This result showed NP brush sampling could collect shedding cells from NP tissue, including tumor cells.

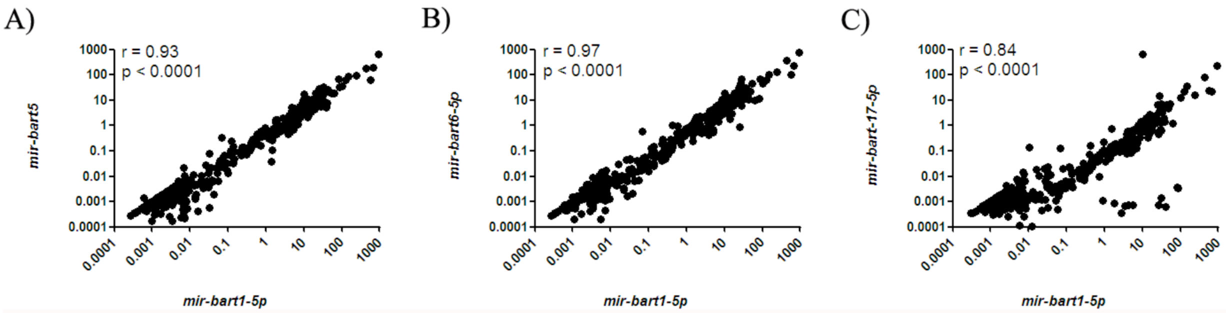

Supplementary Figure S2: Significant correlations among the four EBV miRNAs were observed. The correlation coefficients of *mir-bart1–5p* with *mir-bart5* **A.** *mir-bart6–5p* **B.** and *mir-bart17–5p* **C.** were 0.93, 0.97 and 0.84, respectively (all with $p < 0.0001$).

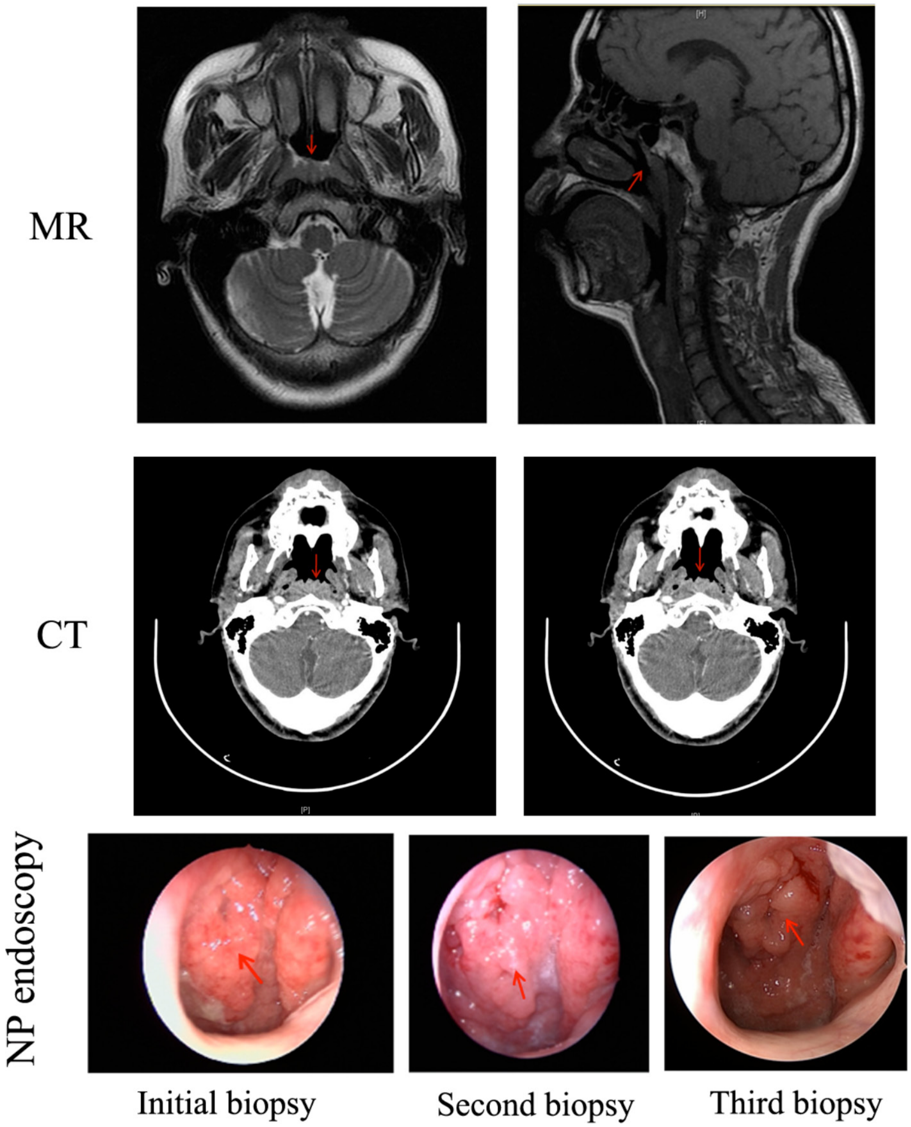

Supplementary Figure S3: CT and MR results, along with endoscopy images, are shown for a representative patient.

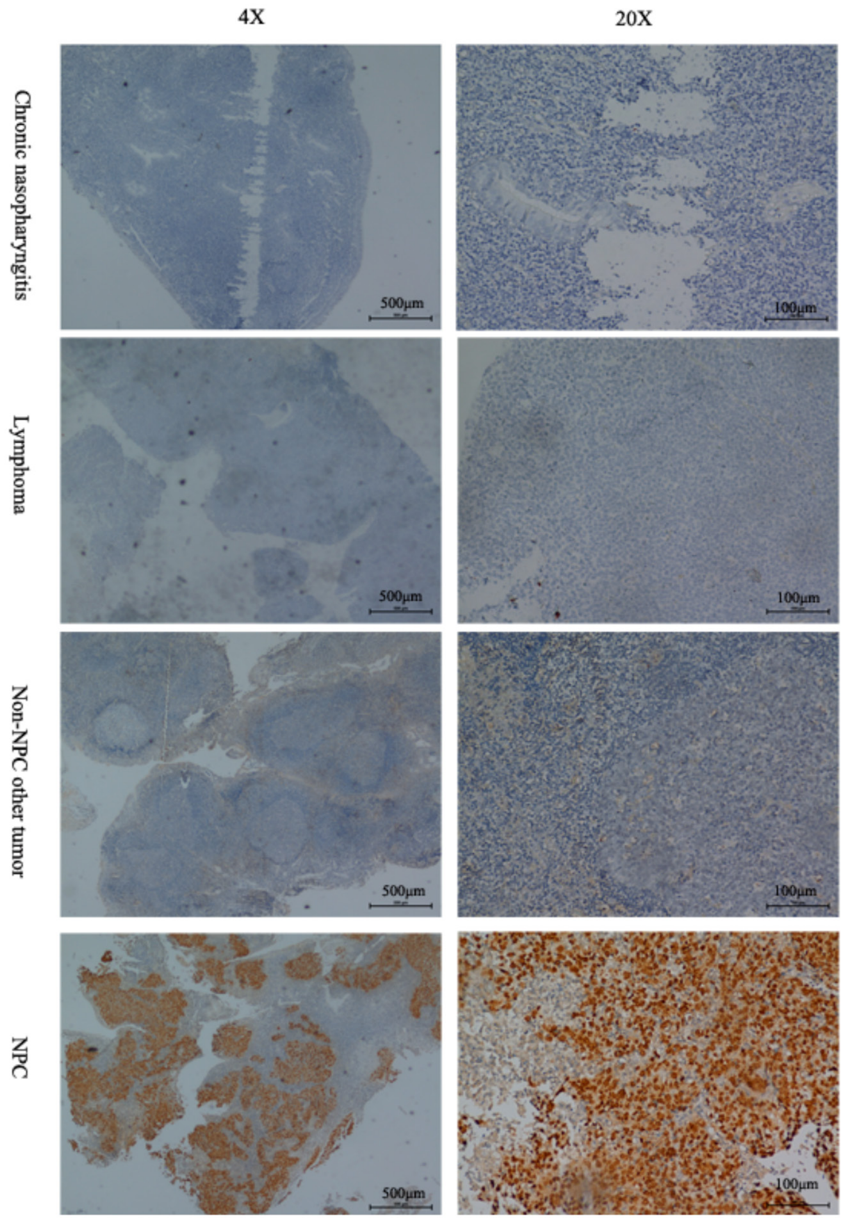

Supplementary Figure S4: Representative results of EBERs *in situ* hybridization using NP tissue slices from different participants. High EBERs signal was only observed in the tissue slices from NPC patients.

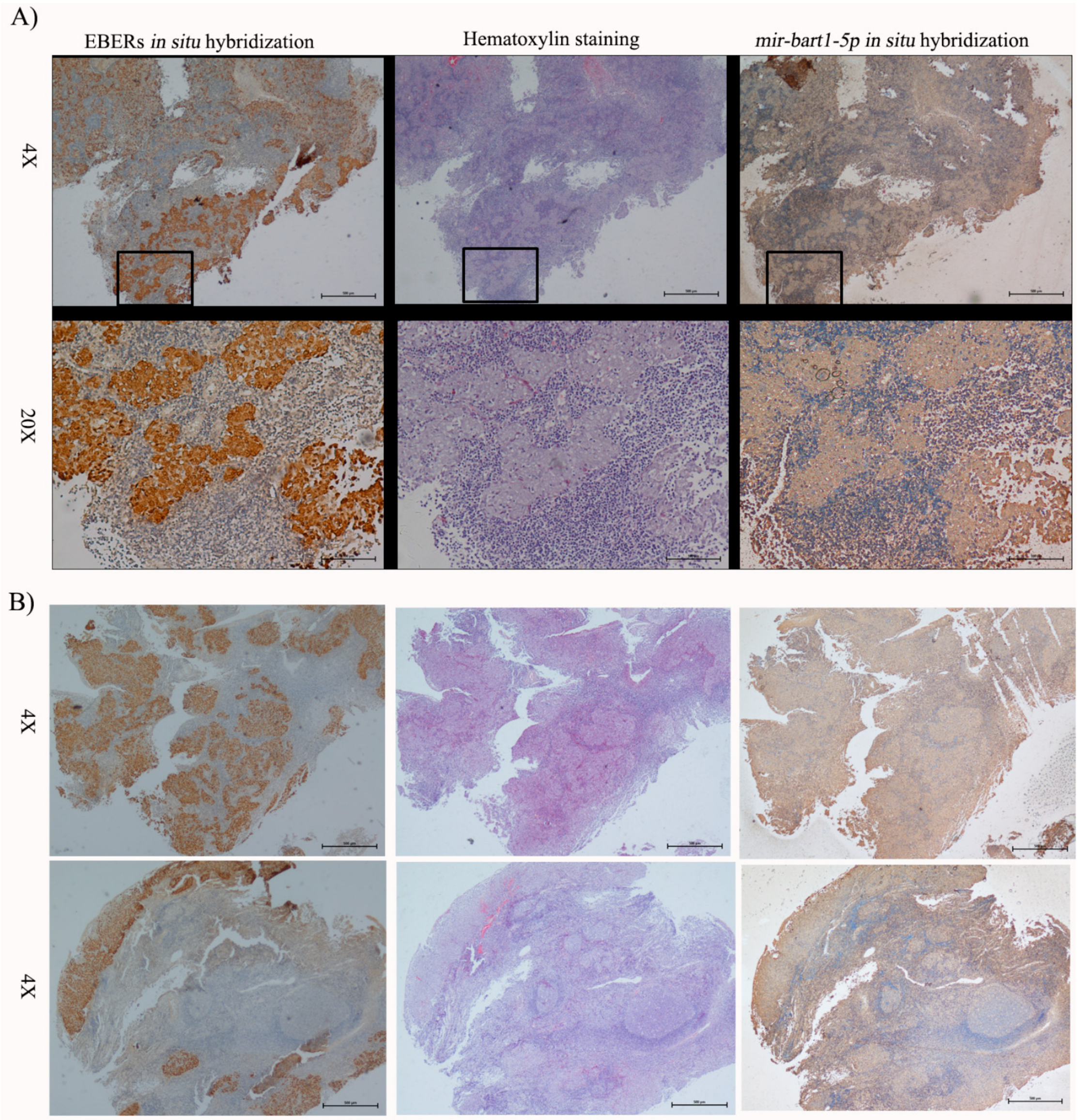

Supplementary Figure S5: Representative results of EBERs and *mir-bart1–5p in situ* hybridization using NP tissue slices with negative *mir-bart1–5p* expression in NP brush samples. The results showed *mir-bart1–5p* and EBERs were both expressed in nasopharyngeal tumor cells **A.** Two representative samples with negative *mir-bart1–5p* expression in NP brush samples **B.**

Supplementary Table S1: The relative expression of EBV miRNAs in tissue samples

EBV miRNAs	Group	Median	Minimum	Maximum	Mann-Whitney test
mir-bart1–5p	NPC	68	0.2	710	P < 0.001
	Controls	0.002	0.00033	0.041	
mir-bart5	NPC	11	0.033	72	P < 0.001
	Controls	0.001	0.00032	0.0093	
mir-bart6–5p	NPC	33	0.058	150	P < 0.001
	Controls	0.003	0.00034	0.044	
mir-bart17–5p	NPC	4.1	0.00038	59	P < 0.001
	Controls	0.001	0.00032	0.0083	

Supplementary Table S2: The relative expression of EBV miRNAs in NP brush samples

EBV miRNAs	Group	Median	Minimum	Maximum	Mann-Whitney test
mir-bart1–5p	NPC	2.05	0.00087	920	P < 0.001
	Controls	0.002	0	0.040	
mir-bart5	NPC	0.84	0.00031	660	P < 0.001
	Controls	0.001	0	0.022	
mir-bart6–5p	NPC	1.08	0.00054	770	P < 0.001
	Controls	0.002	0	0.021	
mir-bart17–5p	NPC	0.12	0.00031	650	P < 0.001
	Controls	0.001	0	0.018	

Supplementary Table S3: Diagnosis of NPC by repeated pathological biopsy and *mir-bart1–5p* expression

Sample ID	*mir-bart1–5p* expression	Number of biopsies
50	0.96	2
62	8.64	2
66	0.05	2
78	1.83	3
81	3.42	2
88	0.23	2
107	0.23	2
125	0.05	2
154	0.08	2
172	1.56	2
181	3.12	2
209	0.26	2
222	0.74	5
228	0.39	2
260	0.06	4
268	6.86	2

Repeated biopsy sampling was conducted in 16 patients before obtaining a correct pathological diagnosis. Levels of EBV ***mir-bart1–5p*** in NP brush samples from these patients were all above the COV.
